# The effect of performance-based financing interventions on out-of-pocket expenses intended to improve access to and utilization of maternal health services in sub-Saharan Africa: protocol for a systematic review and meta-analysis

**DOI:** 10.1186/s13643-022-01990-9

**Published:** 2022-06-30

**Authors:** Miriam Nkangu, Julian Little, Olumuyiwa Omonaiye, Sanni Yaya

**Affiliations:** 1 School of Epidemiology and Public Health, University of Ottawa, Ottawa, Canada; 2 Health Promotion Alliance Cameroon (HPAC), Yaounde, Cameroon; 3 School of Nursing and Midwifery, Centre for Quality and Patient Safety Research, Institute for Health Transformation, Deakin University, Burwood Campus, Melbourne, Australia; 4 Centre for Nursing and Midwifery Research, James Cook University, Townsville, QLD Australia; 5 School of International Development and Global Studies, University of Ottawa, Ottawa, Canada

**Keywords:** Performance-based financing, Out-of-pocket expenses, Access, Health sector, Systematic review, Maternal health services, Antenatal care, Skilled birth delivery, Family planning, Sub-Saharan Africa

## Abstract

**Background:**

Out-of-pocket expenses have been reported as a major barrier to accessing antenatal care and skilled birth delivery in most of sub-Saharan Africa. Performance-based financing (PBF) is one of several strategies introduced in lower- and middle-income countries to strengthen a weak health system. This review aims to synthesize evidence on the effectiveness of PBF interventions implemented with the objective of reducing out-of-pocket expenses and improving access to and utilization of ANC and skilled birth delivery and family planning in sub-Saharan Africa. It will consider evidence across health sectors and identify gaps in the evidence.

**Methods and analysis:**

This protocol is reported according to Preferred Reporting Items for Systematic Review and Meta-Analysis Protocols (PRISMA-P) guideline. The systematic review will apply a three-step strategy to search five databases (CINAHL, PubMed, Ovid Medline, EMBASE, Cochrane.) and grey literature with the help of a librarian. Two independent reviewers will conduct screening to determine eligibility and critical appraisal of selected studies using the risk of bias criteria developed by the Cochrane EPOC Group and the New Castle Ottawa Scale for observational studies. The certainty of evidence for the outcomes will be assessed using “Grades of Recommendation, Assessment, Development, and Evaluation” (GRADE) approach. This review will consider experimental and quasi-experimental study designs and observational studies. Studies published in English and French language(s) will be included. Studies published since the introduction of PBF in sub-Saharan Africa will be included. Data will be collected on each item that contributes to out-of-pocket expenses. This review will adopt the Multiple Dimensions of Access Framework to organize the findings.

**Discussion:**

This systematic review will support evidence-informed data for the performance-based financing community and government by identifying, describing, and assessing the impact of performance-based financing interventions on out-of-pocket expenses in promoting access and utilization of ANC, skilled birth delivery, and family planning across health sectors.

**Systematic review registration:**

This review has been registered with PROSPERO, Registration number CRD42020222893.

**Supplementary Information:**

The online version contains supplementary material available at 10.1186/s13643-022-01990-9.

## Background

Health financing is a building block of the healthcare system, directed towards improving health outcomes, protecting people financially against the catastrophic or impoverishing cost of care, and ensuring that healthcare services satisfy the population in need [1]. Health financing is important in the efforts toward improving health systems and targeting universal health coverage (UHC) [2]. One of the key elements of UHC is to improve the efficient use of financial resources [3]. Financial aspects have been reported as a major barrier to accessing antenatal care (ANC) and skilled birth delivery in most of sub-Saharan Africa [4–6]. These financial barriers among other factors have contributed to high rates of maternal mortality of up to 600/100,000 lives birth in most countries in this region [4–7]. In an attempt to strengthen the weak health systems and address some of the above-mentioned problems, Performance-based financing (PBF) was implemented in many African countries with a focus on maternal health services [7]. This is done by paying providers according to the quality and quantity of services delivered for predetermined conditions [2–4, 7, 8].

Several terms have been used to describe PBF [6]. For instance, result-based financing (RBF) is utilized in some instances to describe PBF; however, RBF includes all incentives approaches for both demand and supply side perspective and may target any level of the health system based on the design. [8, 9]. Pay-for-performance is synonymous with RBF and is often used in higher-income countries such as Canada, New Zealand, the United Kingdom (UK), and the United States (US) [9, 10]. Performance-based contracting (PBC) involves the use of non-governmental organizations, as for example in Afghanistan; it is the same as PBF, but the difference is that, while PBF contracts with health facilities (either public or private), PBC contracts with non-governmental organizations [9, 11]. PBF is a supply-side strategy, but to enhance their effectiveness, some PBF programs also include demand-side strategies that use vouchers or equity funds such as in Nigeria [9, 11] or social health insurance schemes as in Burundi [7, 8, 11, 12]. This review will use PBF as the operational term.

The focus of PBF is on maternal and child health services, with the goal to improve the quality and equity of coverage [2, 3, 10–14]. These equity elements include, but are not limited to, “subsidizing user fees and paying providers more for services delivered in poor areas to reduce out-of-pocket (OOP) expenditures, enhance financial protection and increase the utilization of maternal services to reduce inequity” [9, 11, 12, 15, 16]. OOP expenditures are defined as the direct payments made by individuals to healthcare providers at the time-of-service utilization [17]. This excludes any prepaid payment, notably insurance [17]. These OOP expenditures (also known as user fees) are usually categorized into formal and informal fees [9, 17]. Formal fees are payments that are officially recognized and stipulated by the health facility and include fees for medications, contraceptives, laboratory tests, provider services, facility use, and other expenses such as transportation [17]. Informal or unofficial payments are payments made more than official fees, also called “under the table” payments [11, 17]. PBF is a supply-side strategy that pays providers incentives to change their behavior and stimulate demand for services, making services affordable [1, 2, 9, 11]. One of the ways through which PBF can influence the use of maternal services is the effect on OOP expenses. Thus, PBF assumes that “subsidizing user fees to improve financial protection by reducing OOP expenditures will reduce inequities” [9]. This component of PBF equity design constitutes one of the areas where PBF intersects with UHC; that is, “expanding the coverage of health services for the general population, especially for the poorest” [9].

In some countries, positive impacts of PBF on maternal health services have been reported. For example, in Rwanda, increases in the proportion of women who had skilled birth delivery in health facilities and the quality of prenatal care were reported [18]. In the Democratic Republic of Congo, PBF improved the health provider’s effort to increase targeted service provision [19]. In Cameroon, PBF led to a significant increase in utilization (child and maternal immunization, family planning, HIV testing) [20]. However, limited impact of PBF, and/or potential increased inequity resulting from PBF, have also been reported. Indeed, in Cameroon, there was no impact on skilled birth deliveries and ANC visits [20], and while PBF had an effect on informal fees, there was no impact on formal fees [20]. In Rwanda, although the PBF program did not increase inequities, it did not reduce them either [21, 22]. In Tanzania, a “potential pro-poor effect” on institutional deliveries was found among women in the poorest income subgroups [23, 24]. Studies in Rwanda and Burundi reported pro-rich effects [12, 25, 25]. In Burundi, following the implementation of PBF, there was a 4% increase for institutional delivery among the “rich” but no effect for the poor—this difference was statistically significant [12]. In Rwanda, PBF was found to be less effective in reaching the poor and favored the rich on improving access [25]. A Cochrane review on the effectiveness of PBF on the delivery of maternal health services studied several outcomes including ANC and skilled birth delivery and reported a mixed effect on institutional deliveries [26]. Some studies found an increase in institutional delivery, but other studies found no substantial increase [26]. The Cochrane review found both positive and negative effects on the utilization of ANC [26]. The review concluded that based on low-quality studies, there was weak evidence to support any conclusion [26]. However, this review was conducted in 2011 when PBF programs in most countries were still undergoing impact evaluations; since then, some countries have scaled-up and institutionalized PBF. Thus, the Cochrane review requires an update. Our review will be focused on PBF effects on OOP expenses and across health sectors and will update some of the outcomes of the Cochrane review within the context of sub-Saharan Africa.

Health care delivery in most sub-Saharan Africa countries is largely delivered in both public and private sectors. The OOP expenses for maternal health services also vary by health sector. PBF intervention is implemented across all health sectors, but the results are usually reported in an aggregate form without consideration of heterogeneity across health sectors and within the OOP items. A systematic review on the effect of PBF on the quality of maternal services [27], reported on OOP expenditures as a secondary outcome with a focus on consultation fees. The review did not focus on specific components of OOP expenses and did not present results by health sector [27]. A systematic review conducted by James et al., 2020 focused on result-based financing schemes and the evidence on the level on which PBF has been scaled and/or institutionalized for maternal and child health services, but it did not focus on OOP items and health sectors [28].

These OOP items may be impacted differently with the implementation of PBF. However, typically, only OOP expenses overall are reported and the effect of PBF on each OOP component has been overlooked. This constitutes a limitation in understanding where the implementation of PBF may need improvement with a view to reducing out-of-pocket costs, enabling enhanced financial protection and increased use of services.

Moreover, PBF interventions on user fees typically do not treat OOP expenses as an aggregate variable. Instead, each item that contributes to OOP expenses, for example, laboratory, echography, contraception, medication, and consultations fees, can be incentivized and/or purchased in different ways across health facilities and sectors. Given the important role that financial barriers play in accessing ANC and skilled birth delivery [4–6], and the effect of PBF on each of these OOP items, a major gap is the lack of an evidence synthesis on the effects of PBF on different components of OOP expenses, in particular, to improve access to ANC, family planning and skilled birth delivery across health sectors. Identifying the role those financial barriers play in accessing ANC and skilled birth delivery and family planning, and the role that each OOP item plays in determining the ultimate cost for ANC visits and skilled birth delivery, which also varies by health sector, will allow for a better and broader understanding of where the gaps are, and the system’s failures related to OOP expenses. Such understanding will inform country-level policies on ANC and skilled birth delivery adopted to achieve universal healthcare coverage and the PBF community at large on the interventions towards OOP expenses to improve equity.

This review will synthesize the effect of PBF on OOP expenses on access to and utilization of maternal health services across health sectors in sub-Saharan Africa. The aim is to produce a systematic review that identifies, describes, and characterizes the gaps on the effectiveness of implemented PBF interventions intended to change OOP expenses and increase access to and utilization of ANC, skilled birth delivery, and family planning across health sectors in sub-Saharan Africa. The review will extract and describe each element that contributes to OOP expenses and characterize the gaps across sectors. If the review finds enough evidence to generate the effectiveness of those OOP interventions across health sectors, then, a meta-analysis will be conducted.

### Defining access and measures

Several factors have been reported to affect the uptake of maternal services which can be categorized into structural barriers, financial barriers, and personal and cultural barriers [29]. Access to health care is a complex phenomenon defined in several ways. Andersen and colleagues defined access as the “actual use of personal health services and everything that facilitates or impede[s] their use” and as “not merely defined by the availability of services or health facility or resources but rather, if these services are actually being used by those who need them” [29]. The framework on access described by Andersen and colleagues identifies both contextual and individual factors as major determinants in improving access [29]. These contextual and individual factors are categorized into predisposing, enabling, and need characteristics. This review will focus on the contextual and individual enabling characteristics as they directly facilitate or impedes the use of services [29]. The rationale for using this framework is based on the interrelationship between financial aspects (OOP expenses) and the mediating role they have on access to and the utilization of services and the desired outcomes [29].

According to the model, contextual enabling characteristics include the health policy, financing mechanism, and organizational factors [29]. The health policy in place is considered as a policy instrument and a starting point to improve access, given its political importance [29]. Performance-based financing does not only raise political issues as a health reform, but also a financing mechanism intended to improve financial accessibility. Secondly, the financing aspect includes the available resources that can potentially pay for health services, and incentives to purchase or provide health services [29]. In this context, the targeted PBF policies (or incentives) on user fees or OOP for antenatal care, skilled birth delivery, and family planning to improve access, and lastly, the organizational factor which defines PBF considerations on OOP expenses and how it is applied across all health facilities in both private and public health sectors and the targeted communities or population. The individual enabling characteristics include financing and organizational characteristics. The financing characteristics are the cost of care or “effective prices” and the availability of income for individuals to be able to purchase the services [29]. Organizational characteristics are the support in terms of available means of transportation for individuals and/ or other support mechanisms within the organization [29]. According to these authors, the financial barrier is one of the components of inequity while access is a measure of utilization [29]. They categorized access into six dimensions to guide policy on improving and monitoring access. These dimensions include potential, realized, equitable, inequitable, effective, and efficient access as described in Table 1. It should be noted that we have included an adapted contextual description column in Table 1 to describe the approach we would use to explain the dimensions of access within the context of this review.

**Table 1:** Dimensions of access as defined by Andersen et al.

Dimensions of access by Andersen et al	Definition from Andersen et al	Application in systematic review
Potential access	Health financing policy in place	Implementation of performance-based financing policy
Realized access	Actual utilization of services as a result of the health financing policy	Utilization based on SR outcomes (ANC, skilled birth delivery, and family planning) as reported in the study
Effective access	Improving health status from health service use and is a function of potential and realized access	Changes in the outcomes in terms of utilization as reported in the study, for example, timely use of services to improve health outcomes—attributed to the PBF policy
Equitable access	Ensures distribution of resources based on need	Consideration of equity variables—income groups, place of residence, and contextual differences in resource allocation as reported in the study
Inequitable access	Focused on the process of reducing the influence of social characteristics on the distribution of health services	Consideration of approaches used to address information barriers among population groups as reported in the study
Efficient access	To minimize the cost of improving outcomes as a result of health service use	Cost minimization for specific services (ANC, skilled birth delivery, and/or family planning) to improve utilization and outcome as reported in the study

Source: Adapted from Andersen et al. (2013)

### Theory of change

PBF strives to improve access and the quality of maternal health care with special consideration on equity coverage [9, 11]. PBF assumes that “subsidizing user fees and paying providers more for services delivered in poor areas in order to reduce OOP expenditures, it will enhance financial protection and increase the utilization of maternal services and reduce inequity” [9]. This review builds on the above PBF theory of change by applying Andersen et al.’s contextual and enabling characteristics of the behavioral model on healthcare utilization which assumes that the health policy in place, in this context, PBF policy on OOP has an effect on the delivery of care [29]. The way OOP expenses for ANC, skilled birth delivery, and family planning are defined and implemented across health facilities and health sectors and within rural and urban settings has an impact on access and utilization of ANC, skilled birth delivery, and family planning. These contextual and individual characteristics can influence access in multiple dimensions. Thus, the review will discuss and categorize any changes in the outcome using the multiple dimensions of access as described by Andersen and colleagues [29].

## Methods

### Reporting and registration

This protocol is reported according to the guidelines for preferred reporting items for systematic review and meta-analyses protocols (PRISMA-P 2015) Checklist (see attached supplemental file 1) [30, 31]. The systematic review will use a three-step search strategy with a minimum of 5 databases (CINAHL, PubMed, Ovid Medline, EMBASE, Cochrane.) and grey literature with the help of an information scientist. This review is registered with PROSPERO, the International Prospective Register of Systematic Reviews. Registration number CRD42020222893.

### Inclusion and exclusion criteria

#### Population

The review will include health facilities in both public and private sectors, health care providers, and pregnant women or household members attending antenatal care, skilled birth delivery, and family planning that are being assessed within a PBF program.

#### Intervention(s)

This review will consider studies that evaluate any PBF intervention or strategy specifically designed to target any of the items that constitute OOP expenses as listed in the OOP item list below. PBF interventions can be at the community level, which may involve community health workers and health facilities. Specifically, it will investigate PBF OOP interventions directed at medical services for ANC, skilled birth delivery, and family planning.

Interventions could also be demand-side financing, whereby, incentives are provided to health facilities and/or providers to achieve some level of standard and performance as expected for ANC, skilled birth delivery, and family planning [9, 11]. Any demand-side intervention implemented as part of the PBF program on OOP items to ensure the effectiveness of the program will be considered. Demand-side interventions that were not included as part of the PBF mechanism for OOP or other national interventions will not be considered; they will be reported but will not be included in the synthesis (see Table 2).

**Table 2:** Inclusion and exclusion criteria

Inclusion criteria	Exclusion criteria
Presence of at least one comparison group	Studies with any comparator
Published from 1990 to 2020 since PBF started in the 90 s	
Interventions at facility or provider	PBF intervention policies on OOP without concrete description of implementation at facility level or point-of-care will be included for reporting purposes but will not be included in the final synthesis
Studies in sub–Saharan Africa	Studies in LMIC without disaggregating results separately for sub-Saharan countries will not be included in the final synthesis, but will be reported
Study reporting effect change, or any changes observed as a result of the PBF intervention on OOP and statistic significance of the intervention	Studies that do not disaggregate the reporting of OOP items will not be included in the final synthesis, but they will be reported separately
Study assesses PBF intervention (or demand side) on at least one item that constitutes out-of-pocket expenses	Study is not focused on PBF intervention in relation to out-of-pocket expenses
Study assesses at least one outcome of interest that is ANC, skilled birth delivery, or family planning	Study assesses other maternal services but does not assess any one of the outcomes listed will
The study quantitatively evaluates the effect of any PBF interventions	Study is a qualitative and/or does not evaluate the impact of PBF intervention
Study clearly describes the PBF intervention on the specific OOP expenses and provides details on the outcome	Studies that only describe PBF intervention on OOP without providing effect on the outcomes will be included for reporting purposes but will not be included in the final synthesis

#### Out-of-pocket expenditures items

Out-of-pocket expenses include direct and indirect expenses [17]. Indirect expenses are any form of informal fees while direct expenses are defined as follows [17]:

i) Medication cost includes all pharmaceuticals related expenses on routine drugs during ANCs and skilled birth delivery
ii) Contraception fees relating to fees for services like family planning and contraceptive methods, include all fees paid for contraceptive
iii) Clinical test includes services like laboratory test, X-ray, echography, and laboratory test during ANCs especially ANC1 and or skilled birth delivery
iv) Facility fees are usually collected for registration, consultation, or overnight stays
v) Provider’s fee may relate to specialized care services by health care providers, for example, C-section
vi) Other (may include transportation cost to facility)

We will not have any restrictions for the type of PBF intervention or strategy. There will be no limitations on the length of follow-up of the intervention.

#### Comparators

This review will identify the studies that compared PBF intervention on OOP to an active or non-active comparator, for example, comparators could be standard of care practice, other health interventions or policies that are not directed by PBF, or novel comparators. No eligibility criteria will be made for comparators used.

### Outcomes

The outcome for this review is access to ANC, skilled birth delivery, and family planning, (i) any PBF interventions on out-of-pocket expenses for ANC, skilled birth delivery, and family planning and (ii) any observed or reported changes on ANC, skilled birth delivery, and family planning due to OOP changes as a result of PBF and across health sectors. The secondary outcomes will consider any PBF interventions on OOP on other maternal health indicators like maternal immunization and nutrition. Antenatal care visits will include ANC 1–4 visits and more where applicable. Skilled birth delivery includes community and institutional deliveries performed by an accredited trained professional [1]. Access will be presented using the six dimensions as described in Table 1 above [29].

For the outcome, we will include studies that measure ANC, skilled birth delivery, and the use of family planning methods. These variables are defined above. Studies that will be included will be restricted to sub-Saharan Africa. Skilled birth delivery by a trained professional can be either in the hospital or at home. At this stage, in order to avoid bias, we will not restrict publication year and language. The following will not be included in the study, commentaries, perspectives, expert opinions, conference proceedings, editorials, and book chapters. Publications that do not include full texts will be excluded in the review but will be included in mapping the evidence.

### Study designs

This review will include impact evaluations and observation studies of PBF interventions directed toward OOP for antenatal care, skilled birth delivery, and family planning across health sectors. The following study designs will be considered for this review, experimental and quasi-experimental study designs, and observational studies. Thus, we will include randomized and non-randomized controlled trials, before and after studies, and interrupted time-series studies. For observational studies, we will include prospective and retrospective cohort studies, case–control studies, and cross-sectional studies.

### Search strategy

The review will search five databases (Ovid Medline, CINAHL, PubMed, Cochrane, Embase), including CABI Global Health (Ovid). The search terms will be developed in consultation with an information specialist. We will combine the search terms for PBF and sub-Saharan Africa. We will develop the search criteria initially in Ovid Medline and additional searches will be adapted from the initial search strategy and tailored according to each database. Grey literature will be searched from the following databases: World Bank e-library for PBF impact evaluations, Epistemonikos database, Department for International Development (DfID's) research outputs database, and the ELDIS development database (eldis.org). Citation searches of included studies will be run using Google Scholar. The reference list of all studies selected for critical appraisal will be screened for additional studies. Studies published in English and French language(s) will be included. Studies published since the introduction of PBF in sub-Saharan Africa will be included, that is since the 1990s.

### Data management

The search results will be exported into Endnote for data management, such as duplication removal, and referencing. After removal of duplicates, data will be exported into Covidence [32] for screening and data extraction. Subsequently, data analysis will be conducted, and depending on the possibility of conducting a meta-analysis, data will be exported into the review manager (RevMan) [33] for further analysis.

### Study selection process

The review process (screening, eligibility, inclusion, and meta-analysis) will be conducted independently by the two reviewers using Covidence. The titles and abstracts will be screened by the two independent reviewers using the inclusion criteria for the review. Studies that may meet the inclusion criteria will be retrieved in full during full-text screening. The full text of the selected studies will be retrieved and assessed in detail using the inclusion criteria. Full-text studies that do not meet the inclusion criteria will be excluded and the reasons for exclusion will be listed in the final systematic review report. Papers will be retained for full-text screening when there is uncertainty about the eligibility of a paper at the initial screening stage. The results of the search will be reported in full in the final report and presented in a PRISMA flow diagram. Any disagreements that arise between the reviewers will be resolved through discussion, or with a third reviewer. Understanding of the inclusion and exclusion criteria will be assessed through calibration of a small number of studies strictly adhering to the PICO criteria. Any disagreement will be resolved through consensus, if consensus is not achieved, a third reviewer(co-authors) will be consulted. If a study has multiple publications, the most recent one will be retained. At this stage, language will be limited to French and English. Similarly, at this stage, we will limit the scope to sub-Saharan Africa and we may limit the publication year depending on the nature of interventions and the number of studies.

### Data extraction

**Fig. 1:**
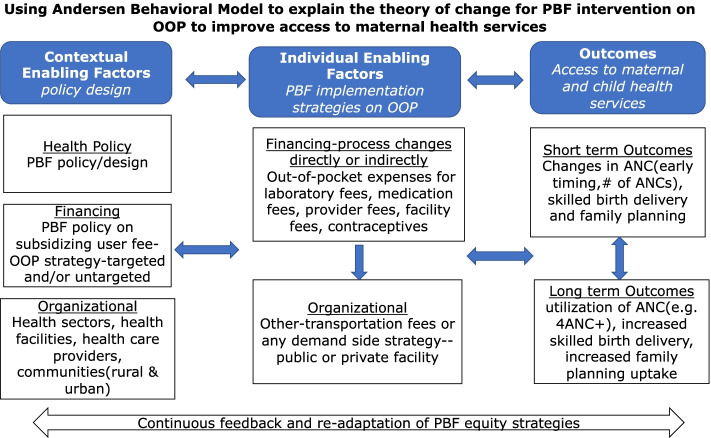
Theory of change

Data will be extracted from papers included in the review using the data extraction tool adapted from the data extraction tool developed by the Cochrane EPOC Group [34]. The data extraction form will be validated by the team on the data variables to be extracted. The data extraction will be done by the two independent reviewers. The data extracted (which will incorporate elements in Fig. 1) will include specific details about study characteristics, study participants, intervention details, study design and methods, outcome details, intervention effects, data analysis, consideration of equity dimensions, category of access measures for outcome variables and context characteristics. See Table 3 for additional information on data extraction. Any disagreements that arise between the reviewers will be resolved through discussion or with a third reviewer. Authors of papers will be contacted to request missing or additional data where required.

**Table 3:** Data extraction tool

Dimensions	Details	Additional explanation as applicable
General information	Authors, year, country of study, language,	
Study characteristics	aim of PBF intervention, PBF program, health financing mechanism before PBF, study design, aim of the study, health system structure (private and public sector), level of scale of PBF by country, data source, methods	
Study participants	Description of study population, characteristics of study participants, recruitment strategies, sample size calculation	
Intervention details	Out-of-pocket items, description of the intervention, intervention groups, number of interventions, and the timing	Any of the 6 out-of-pocket items, medication, laboratory, contraception fee, and by health sectors
Data analysis	Nature of analysis conducted and statistical test, control for confounders	
Outcome details	Type of outcomes, ANCs, skilled birth delivery, family planning	
Intervention effects	Baseline and post PBF results, effect estimates and subgroup analysis estimates, secondary outcome effects	
Equity dimensions	Consideration of equity elements	Any of the following considered in the intervention to reduce OOP, rural or urban setting, occupation, education, income status
Category of access measures for outcomes	Potential access, realized access, equitable access, inequitable access, effective access, efficient access	As described in Table 1
Context characteristics	Political setting, conflict setting, non conflict setting, stage of PBF program (pilot, scale, institutionalized stage)	

Source: Adapted from Cochrane EPOC Group 2017

### Quality assessment and risk of bias

All selected studies will be critically appraised by two independent reviewers at the study level for methodological quality in the review using the risk of bias criteria developed by the Cochrane EPOC Group [34]. This tool is based upon the Cochrane Collaboration risk of bias tool [35]. The tool provides advice on assessing the quality of the methodology for randomized control trials, evaluating risk of bias including randomization, quality of randomization, allocation concealment, level of blinding, masking, and losses to follow-up. Studies will be assessed based on selection bias, performance bias, detection bias, attrition bias, reporting bias, and any other sources of bias. The rating for bias will be reported as “high”, “low” or “unclear” [35]. Any disagreements that arise will be resolved through discussion, or with a third reviewer. The Cochrane Collaboration recommends the same tool can be applied at minimum, to evaluate risk of bias in non-randomized studies [35]. However, the Newcastle Ottawa Scale has been reported to be the most useful for systematic reviews for non-randomized studies [36, 37], but the variations in non-randomized study designs make it difficult to apply [35, 37]. We will critically appraise the studies while applying the above tools and look for potential confounding and evaluate how the authors have adjusted for confounding, and how they have considered the items in reporting observational studies. Studies in which the intervention or outcomes are not well described by the authors, or in which data are not presented in a way that can be extracted, will be excluded; a list of excluded studies, with reasons for exclusion, will be presented as an appendix to the report. Publication bias will be assessed using funnel plots and visual inspection [35]. Any disagreement during risk of bias assessment will be resolved through consensus and if unable to come to a consensus a third reviewer will be consulted.

### Data synthesis

The data that are collected from included studies will be tabulated and summarized narratively. We will begin with a descriptive review (incorporating elements of the theory of change in Fig. 1). We will describe the characteristics of the included studies which will include the population, the types of studies, PBF program, objectives, health sectors and health financing mechanism, country, methods, and main results. PBF intervention strategies that have been implemented to improve OOP expenses will be categorized and described according to each OOP item—medication, clinical test, contraception fees, provider fees, facility fees, and other fees. A narrative description and structured synthesis will be used to present the intervention effect on the outcomes on access to ANC, skilled birth delivery, and family planning using the dimensions of access as defined above.

Papers will, where possible, be pooled for meta-analysis using RevMan [33, 35]. The outcomes results can be presented as immediate change and/or change in trend before-and-after PBF. Effect sizes will be expressed as either odd ratio (for dichotomous data) and standardized mean differences (for continuous data) and their 95% confidence intervals [35, 36]. Heterogeneity will be assessed through visual inspection of forest plot and statistically using the standard chi-square and *I* square tests (*I*^2^) [38, 39]. The choice of model will be random effect model as we expect that most of the studies will present a heterogenous population. Subgroup analyses will be conducted where there is sufficient data to investigate heterogeneity across health sectors and groups. If we find low-quality studies or studies that are evidently different from the rest, a sensitivity analysis will be conducted. In the case of a missing statistical data, we will contact the authors by using the corresponding address. Authors will be contacted, maximum twice, before we conclude as a missing data. Where statistical pooling is not possible, the findings will be presented in narrative form including tables and figures to aid in data presentation where appropriate. A funnel plot will be generated to assess publication bias if there are 10 or more studies included in a meta-analysis [35]. If there are enough studies with quantitative measures on ANC and skilled birth delivery and consistency in the direction of effects with low variation, a meta-analysis will be conducted.

The certainty of evidence in aggregate for all the outcomes will be assessed using “Grades of Recommendation, Assessment, Development, and Evaluation” (GRADE) [35, 39]. We will rate the certainty of the evidence using the summary of findings table and assess risk of bias, inconsistency, indirectness, imprecision, and publication bias for the outcomes [35, 39, 40]. We will use the Cochrane EPOC Group summary of findings table to present the rating in GRADE [40].

### Interpretation and reporting of review findings

We will use the PRISMA flow chart to present the screening process, giving justifications for exclusions at each level of the flow chart. The synthesized evidence will be presented according to clinical and methodological components, as well as presenting summary narratives and forest plots. We will apply the AMSTAR 2 tool—a critical appraisal tool used in assessing the methodological quality of systematic reviews [41]—as we develop our work to ensure that we maximize quality. The final review will be reported according to the PRISMA guidance [31].

## Discussion

With the rise of PBF as a health reform in health system strengthening and the introduction of PBF equity instruments as a strategy towards achieving UHC. This systematic review will support evidence-informed data for the PBF community and government by identifying, describing, and assessing the impact of PBF interventions on OOP expenses in promoting access and utilization of ANC and skilled birth delivery across health sectors. This review will synthesize the evidence on the effect of performance-based financing on OOP expenses which is one of the instruments listed in the PBF equity program and provide recommendations on access and utilization of antenatal care, skilled birth delivery, and family planning across health sectors in sub-Saharan Africa. The review will discuss the findings in relation to the multiple dimensions of access and characterize the gaps to inform policy and research.

## Limitations

This review will only consider studies published in English and French. This review may also suffer from publication bias given that most of the existing literature may include mostly studies that report positive outcomes [27].

## Supplementary Information

**Additional file 1.**

**Additional file 2.**

## Data Availability

Not applicable.

## Abbreviations

OOP: Out-of-pocket
ANC: Antenatal care
GRADE: Grade Recommendation Assessment Development and Evaluation
PRISMA-P: Preferred Reporting Items for Systematic Review and Meta-Analysis Protocol
AMSTAR: A critical appraisal tool used in accessing the methodological quality of the systematic reviews
