# Supplementary material for: The effect of performance-based financing interventions on out-of-pocket expenses intended to improve access to and utilization of maternal health services in sub-Saharan Africa: protocol for a systematic review and meta-analysis
Source: Syst Rev. 2022 Jun 30;11:133. doi: 10.1186/s13643-022-01990-9 (PMC9248099; doi:10.1186/s13643-022-01990-9)
Supplement: Supplementary file 1 — Additional file 1. [file 13643_2022_1990_MOESM1_ESM.pdf]

Database: Ovid MEDLINE(R)
Search Strategy:
----

1 africa/ or exp "africa south of the sahara"/
2 (Afghanistan or Albania or Algeria or Angola or Argentina or Armenia or Armenian or Azerbaijan or Benin or Botswana or Burkina Faso or Burkina Fasso or Upper Volta or Burundi or Urundi or Cameroon or Cameroons or Cameron or Camerons or Cape Verde or Central African Republic or Chad or Comoros or Comoro Islands or Comores or Mayotte or Congo or Zaire or Cote d'Ivoire or Ivory Coast or Djibouti or French Somaliland or East Timur or Egypt or United Arab Republic or Eritrea or Ethiopia or Gabon or Gabonese Republic or Gambia or Ghana or Guinea or Kenya or Lesotho or Liberia or Libya or Macedonia or Madagascar or Malaya or Malawi or Mali or Mauritania or Mauritius or Mozambique or Niger or Nigeria or Rwanda or Ruanda or Sao Tome or Senegal or Seychelles or Sierra Leone or Somalia or Sudan or Swaziland or South Africa or Tanzania or Togo or Togolese Republic or Tonga or Uganda or western Sahara or West Bank or Zambia or Zimbabwe).ti,ab,kw.
3 1 or 2
4 reimbursement.mp. [mp=title, abstract, heading word, drug trade name, original title, device manufacturer, drug manufacturer, device trade name, keyword, floating subheading word, candidate term word]
5 reimbursement.ti,ab,kw.
6 (pay* adj2 performance*).ti,ab,kw.
7 ((incentiv* or compensat* or reimburs*) adj2 plan?).ti,ab,kw.
8 (conditional adj2 (pay* or transfer?)).ti,ab,kw.
9 ((result* or perform* or output* or out put*) adj2 (financ* or fund* or pay* or disburs* or fee?)).ti,ab,kw.
10 ((pay* or monetar* or economic* or financ*) adj2 (compensation or incentive? or reward* or bonus or bonuses)).ti,ab,kw.
11 (financial support* or remunerat* or salar* or earnings).ti,ab,kw.
12 (performance adj2 financing*).ti,ab,kw.
13 4 or 5 or 6 or 7 or 8 or 9 or 10 or 11 or 12
14 3 and 13
